# Effectiveness of endoscopic ultrasound (EUS)‐guided choledochoduodenostomy vs. EUS‐guided gallbladder drainage for jaundice in patients with malignant distal biliary obstruction after failed endoscopic retrograde cholangiopancreatography: Retrospective, multicenter study (GALLBLADEUS Study)

**DOI:** 10.1111/den.14750

**Published:** 2024-02-21

**Authors:** Antoine Debourdeau, Jules Daniel, Ludovic Caillo, Eric Assenat, Martin Bertrand, Thomas Bardol, François‐Régis Souche, Philippe Pouderoux, Romain Gerard, Diane Lorenzo, Jean‐François Bourgaux

**Affiliations:** ^1^ Hepatogastroenterology Department Nimes University Hospital, University of Montpellier Nimes France; ^2^ Surgery Department Nimes University Hospital, University of Montpellier Nimes France; ^3^ Hepatogastroenterology Department Montpellier University Hospital, University of Montpellier Montpellier France; ^4^ Surgery Department Montpellier University Hospital, University of Montpellier Montpellier France; ^5^ Hepatogastroenterology Department Lille University Hospital, Lille University Lille France; ^6^ Hepatogastroenterology Department Beaujon University Hospital, Paris Cité University Paris France

**Keywords:** biliary drainage, endoscopy, EUS, gallbladder, pancreatic cancer

## Abstract

**Objectives:**

The aim of this study was to compare endoscopic ultrasound‐guided choledochoduodenostomy (EUS‐CDS) vs. EUS‐gallbladder drainage (EUS‐GBD) in cases of failed endoscopic retrograde cholangiopancreatography (ERCP) for jaundice resulting from malignant distal biliary obstruction (MDBO).

**Methods:**

This multicenter retrospective study included patients with obstructive jaundice secondary to MDBO who underwent EUS‐GBD or EUS‐CDS with lumen‐apposing metal stents after failed ERCP. The primary end‐point was clinical success rate. Secondary end‐points were technical success, periprocedural adverse events rate (<24 h), late adverse events rate (>24 h), overall survival, and time to recurrent biliary obstruction.

**Results:**

A total of 78 patients were included: 41 underwent EUS‐GBD and 37 underwent EUS‐CDS. MDBO was mainly the result of pancreatic cancer (*n* = 63/78, 80.7%). Clinical success rate was similar for both procedures: 87.8% for EUS‐GBD and 89.2% for EUS‐CDS (*P* = 0.8). Technical success rate was 100% for EUS‐GBD and 94.6% for EUS‐CDS (*P* = 0.132). Periprocedural morbidity (<24 h) rates were similar between both groups: 4/41 (9.8%) for EUS‐GBD and 5/37 (13.5%) for EUS‐CDS (*P* = 0.368). There was a significantly higher rate of late morbidity (>24 h) among patients in the EUS‐CDS group (8/37 [21.6%]) than in the EUS‐GBD group (3/41 [7.3%]) (*P* = 0.042). The median follow‐up duration was 4.7 months. Overall survival and time to recurrent biliary obstruction did not significantly differ between the groups.

**Discussion:**

After failed ERCP for MDBO, EUS‐GBD and EUS‐CDS show comparable clinical success rates and technical success. EUS‐GBD appears to be a promising alternative for MDBO, even as a second‐line treatment after failed ERCP. Further studies are needed to validate these findings and compare the long‐term outcomes of EUS‐GBD and EUS‐CDS.

## INTRODUCTION

Endoscopic retrograde cholangiopancreatography (ERCP) remains the principal treatment modality for jaundice stemming from malignant distal biliary obstruction (MDBO). Nevertheless, a failure rate of 10–20% has been documented for this condition. Such failures can be attributed to multiple variables such as duodenal stenosis, tumor encroachment at the papilla, duodenal stenting, or complexities in biliary cannulation.^1^, ^2^ In the case of failed ERCP, endoscopic ultrasound‐guided (EUS) biliary drainage has shown better efficacy and reduced morbidity than radiology‐guided percutaneous drainage with reports of >90% success and five times lower postprocedural adverse events (odds ratio 0.23; confidence interval [CI] 0.12–0.47).^3^, ^4^ The feasibility of EUS biliary drainage has been drastically improved since the advent of the electrocautery‐enhanced lumen‐apposing metal stents (EC‐LAMS). Indeed, EC‐LAMS have allowed significant development of biliary drainage via EUS‐guided choledochoduodenostomy (EUS‐CDS).

Several studies have demonstrated the feasibility of EUS‐CDS with high efficacy (>85%).^5^, ^6^, ^7^, ^8^ However, EUS‐CDS has several limitations. First, the procedure may be more challenging in 25% of cases because of anatomical factors (common bile duct [CBD] diameter <12 mm or location too deep, duodenal wall thickness > 10 mm).^9^ Second, LAMS dysfunctions are not rare and can occur in one third (32%) of patients during long‐term follow‐up.^10^ This could be associated with various factors, including sump syndrome, tumor invasion, or compression on the biliary side.^10^, ^11^

Endoscopic ultrasound‐guided gallbladder drainage (EUS‐GBD) using EC‐LAMS is feasible and has been well described in cases of cholecystitis. The procedure has shown high technical success, high efficacy, and reduced morbidity (16–41%) compared with radiology‐guided gallbladder drainage among selected patients with cholecystitis and elevated surgical risk. Adverse events were essentially bile leakage, bleeding, and pleural effusion.^12^, ^13^, ^14^ However, except for cases with cholecystitis, EUS‐GBD can also be performed for MBDO with robust outcomes (100% technical success rate and 85% clinical success rate).^15^ Nevertheless, these data are from studies on small patient numbers, and no comparative studies have been performed on patients with MBDO. Moreover, EUS‐GBD has always been previously described as a “rescue procedure” and never as a second‐line treatment after failed ERCP. The aim of our study here was to retrospectively evaluate EUS‐GBD and EUS‐CDS procedures in cases of failed ERCP for jaundice resulting from MBDO.

## METHODS

### Design

This retrospective, multicenter study (four French tertiary endoscopy centers) included patients with obstructive jaundice secondary to MDBO. All consecutive patients who underwent biliary drainage by EUS‐GBD or EUS‐CDS after failed ERCP between July 2018 and July 2022 were included. Patients were followed‐up until September 31, 2022, or until death. The study was approved by Montpellier University Hospital ethics committee (institutional review board: IRB‐MTP_2022_07_202201185). The study was carried out in accordance with the Declaration of Helsinki.

### Procedures

Endoscopic ultrasound‐guided biliary drainage was performed after failed ERCP. Failed ERCP was defined by an inaccessible papilla, unsuccessful biliary cannulation within 20 min after procedure start, or failure of ERCP in a previous procedure. All procedures were performed with a therapeutic linear echoendoscope. An EC‐LAMS was then placed (Hot AXIOS stent; Boston Scientific, Marlborough, MA, USA) under deep sedation (propofol) or under general anesthesia with endotracheal intubation. The choice between EUS‐CDS and EUS‐GBD was according to the operator's discretion and depended on several factors: the size of the CBD and gallbladder, cystic duct patency, distance between the puncture target and digestive wall, and the presence of duodenal stenosis. All patients were given prophylactic intravenous antibiotics that were discontinued after the procedure in the absence of clinical sepsis.

### EUS‐CDS procedure

All EUS‐CDS procedures were performed as previously described by Jacques *et al*.^5^ with a 6 × 8 mm Hot AXIOS stent. The dilated CBD was directly punctured using a pure cut current. A guidewire was preloaded according to the operator's discretion.

### EUS‐GBD procedure

Endoscopic ultrasound‐guided gallbladder drainage procedures used Hot AXIOS stents of varying dimensions (6 × 8, 10 × 10, 15 × 10 mm). Informed consent was obtained from all participants. Procedures took place in fluoroscopy‐equipped rooms using CO_2_ insufflation. The supervising endoscopists were experts in both ERCP and EUS‐BD. Depending on local protocols, deep sedation was achieved through propofol, with anesthesiological support or under general anesthesia. Cystic duct patency was assessed either intraoperatively or through preoperative imaging; subjects with tumor‐free cystic ducts were included. Patients who had undergone cholecystectomy or who had tumor invasion of the cystic duct were rejected for this procedure. A therapeutic linear array echoendoscope facilitated gallbladder visualization from the distal antrum or duodenal bulb, and color Doppler ruled out vascular interposition. The gallbladder was punctured through the lesser curvature of the antrum using the long‐route technique to optimize stabilization of the scope with either a freehand or wire‐guided technique. For the latter, a 19G fine‐needle aspiration needle was used for positioning, followed by a 0.025 inch hydrophilic‐tipped guidewire placement. Successful Hot AXIOS stent deployment was confirmed by observing bile outflow into the stomach, optionally verified by contrast agent to ensure gallbladder and biliary tree communication.

### Definitions: End‐points

The primary end‐point was comparison of the clinical success rates between patients who underwent EUS‐GBD vs. EUS‐CDS. Clinical success was defined by a >50% decrease in total bilirubin levels at day 7 or normalization at day 28 (<48 μmol/L). Secondary end‐points were as follows:

- Technical success: Adequate LAMS placement between the biliary tract and the foregut with visualization of bile flow.
- Early adverse events: Any adverse events occurring during the procedure or within 24 h after.
- Delayed adverse events: Any biliary adverse events occurring after 24 h after the procedure.
- Significant adverse events was defined by >grade II of the AGREE classification of adverse events.^16^
- Rates of overall survival and time to recurrent biliary obstruction.
- LAMS dysfunction: any biliary complication related to LAMS dysfunction. LAMS dysfunctions were classified according to the classification described by Vanella *et al*.^10^

Early and late adverse events were classified according to the AGREE classification.^16^

#### Duodenal stenosis subgroup analysis

We conducted a comparison of the EUS‐GBD and EUS‐CDS groups among patients presenting with duodenal stenosis, focusing on clinical success, early adverse events, and late adverse events.

### Statistical analysis

Continuous variables are expressed as mean (±SD) for normally distributed data and as median with interquartile range for nonnormally distributed data. Categorical outcomes are presented as absolute and relative (%) frequencies. We created two patient groups according to the drainage procedure (EUS‐GB and EUS‐CDS). Group comparability was assessed by comparing baseline demographic data and duration of follow‐up. The normality of continuous data was assessed using the Shapiro–Wilk test and the heteroscedasticity of continuous data was assessed using the Levene test. For normally distributed continuous outcomes, comparisons were made using ANOVA or Welch's tests. In cases in which continuous data did not follow a normal distribution, the Kruskal–Wallis test was used for comparisons across three or more groups, and the Mann–Whitney *U*‐test was used for comparisons between two groups. The alpha risk was set to 5% and two‐tailed tests were used. We used the Kaplan–Meier method to estimate survival probabilities with their 95% pointwise CIs. The log‐rank nonparametric test for survival distributions was used to compare survival between EUS‐GBD and EUS‐CDS groups. The alpha risk was set to 5% and statistical significance was defined as *P* < 0.05 for all tests. Statistical analyses were performed with EasyMedStat (version 3.24; www.easymedstat.com).

## RESULTS

### Study population

This multicenter study included 78 patients (mean age, 71 years [range, 33–95]; 39 [50%] women). The EUS‐GBD group included 41 (53%) patients and the EUS‐CDS group included 37 (47%) patients (Table 1).

**Table 1:** Baseline patient characteristics

Variable	EUS‐GBD, *N* = 41	EUS‐CDS, *N* = 37
Sex		
Male	22 (53.7%)	17 (45.9%)
Female	19 (46.3%)	20 (54.1%)
	*N* = 41	*N* = 37
Age at inclusion (years)	70.16 (±11.06)	72.62 (±12.25)
	95% CI 66.67; 73.65	95% CI 68.53; 76.7
	Range 51.68; 95.43	Range 33.34; 93.7
	*N* = 41	*N* = 37
World Health Organization performance status score		
0	3 (7.3%)	2 (5.4%)
1	15 (36.6%)	9 (24.3%)
2	12 (29.3%)	10 (27.0%)
3	10 (24.4%)	14 (37.8%)
4	1 (2.4%)	2 (5.41%)
	*N* = 41	*N* = 37
ASA		
1	0 (0.0%)	2 (5.4%)
2	14 (34.2%)	7 (18.9%)
3	23 (56.1%)	27 (73.0%)
4	4 (9.8%)	1 (2.7%)
	*N* = 41	*N* = 37
Etiology of the biliary obstruction		
Pancreatic adenocarcinoma	31 (75.6%)	32 (88.9%)
Cholangiocarcinoma	1 (2.4%)	1 (2.8%)
Metastatic lymphadenopathy	3 (7.3%)	1 (2.8%)
Ampullary carcinoma	3 (7.3%)	2 (5.6%)
Other	3 (7.3%)	0 (0.0%)
	*N* = 41	*N* = 36
Disease stage		
Operable	3 (7.3%)	5 (13.5%)
Borderline	7 (17.1%)	4 (10.8%)
Locally advanced	8 (19.5%)	8 (21.6%)
Metastatic	23 (56.1%)	20 (54.1%)
	*N* = 41	*N* = 37
Total bilirubin (μmol/L)	196.12 (±115.87)	240.19 (±143.0)
	Range 11.0; 543.0	Range 60.0; 694.0
	*N* = 38	*N* = 36
Duodenal stenosis		
Yes	17 (41.5%)	18 (48.7%)
No	24 (58.5%)	19 (51.4%)
	*N* = 41	*N* = 37
Ascites		
Yes	9 (22.0%)	12 (32.4%)
No	32 (78.1%)	25 (67.6%)
	*N* = 41	*N* = 37
Expected therapeutic plan following biliary drainage		
Surgery	3 (7.3%)	5 (13.5%)
Chemotherapy	23 (56.1%)	23 (62.2%)
Supportive care	15 (36.6%)	9 (24.3%)
	*N* = 41	*N* = 37
Cause of ERCP failure		
Papilla invasion	6 (14.6%)	11 (29.7%)
Duodenal stenosis	12 (29.3%)	14 (37.8%)
Difficult biliary cannulation	23 (56.1%)	12 (32.4%)
	*N* = 41	*N* = 37

ASA, American Society of Anethesiologits Score; ERCP, endoscopic retrograde cholangiopancreatography; EUS‐CDS, endoscopic ultrasound‐guided choledochoduodenostomy; EUS‐GBD, endoscopic ultrasound‐guided gallbladder drainage.

### Procedure

Common bile duct diameter was significantly larger in the EUS‐CDS group (16 ± 5 mm) than in the EUS‐GBD group (13 ± 3 mm; *P* < 0.001). In all EUS‐CDS procedures, a 6 × 8 mm LAMS was used. In EUS‐GBD, 63.4% had a 15 × 10 mm LAMS, 24.4% had a 10 × 10 mm LAMS, and 12.2% had a 6 × 8 mm LAMS (Table 2).

**Table 2:** Comparisons of technical aspects and outcomes between endoscopic ultrasound‐guided gallbladder drainage (EUS‐GBD) and endoscopic ultrasound‐guided choledochoduodenostomy (EUS‐CDS) groups

Variable	EUS‐GBD, *N* = 41	EUS‐CDS, *N* = 37
Common bile duct diameter (mm)	13.47 (±3.37) Range 8.0; 24.0	18.19 (±4.58) Range 10.0; 30.0
LAMS diameter		
6 × 8 mm	5 (12.2%)	36 (97.3%)
10 × 10 mm	10 (24.4%)	0 (0.0%)
15 × 10 mm	26 (63.4%)	1 (2.7%)
	*N* = 41	*N* = 37
Double‐pigtail stent		
Yes	2 (4.9%)	6 (20.7%)
No	39 (95.1%)	23 (79.3%)
	*N* = 41	*N* = 29
Technical success		
Yes	41 (100.0%)	35 (94.6%)
No	0 (0.0%)	2 (5.4%)
	*N* = 41	*N* = 37
Clinical success		
Yes	36 (87.8%)	33 (89.2%)
No	5 (12.2%)	4 (10.8%)
	*N* = 41	*N* = 37

LAMS, lumen‐apposing metal stent.

### Primary end‐point

The clinical success rate was similar for both groups; 36/41 (87.8%) patients in the EUS‐GBD group vs. 33/37 (89.2%) patients in the EUS‐CDS group (*P* = 0.8, Table 2). Clinical failure was from persistent liver failure in three (3.8%) patients, progression of the disease in three (3.8%), drainage failure in two (2.6%), and early LAMS obstruction in one (1.3%) patient.

At day 7 postprocedure, the reduction in bilirubin levels was lower in the EUS‐GBD group 50.7% (±25.45) vs. 62.0% (±21.67) in the EUS‐CDS group (*P* = 0.039). At day 30, the bilirubin lowering rate was 68.4% (±32.25) in the EUS‐GBD group vs. 81.7% (±14.38) in the EUS‐CDS group (*P* = 0.07). However, the rate of initiation of chemotherapy postdrainage was similar between the two groups: 47.37% in the EUS‐GBD group and 58.33% in the EUS‐CDS group (*P* = 0.477).

### Secondary end‐points (Table 3)

**Table 3:** Outcomes after endoscopic ultrasound‐guided biliary drainage

Variable	EUS‐GBD, *N* = 41	EUS‐CDS, *N* = 37	*P*‐value
Overall adverse events			
Severity of adverse events (AGREE classification)			0.43
Grade I. Adverse events without the need for pharmacologic treatment or endoscopic, radiologic, or surgical interventions	0 (0.0%)	0 (0.0%)	–
Grade II. Adverse events requiring pharmacologic treatment with drugs other than those allowed for grade I adverse events	2 (33.3%)	1 (10.0%)	–
Grade IIIa. Adverse events requiring endoscopic or radiologic intervention	3 (50.0%)	3 (30.0%)	–
Grade IIIb. Adverse events requiring surgical intervention	1 (16.7%)	4 (40.0%)	–
Grade IVa. Adverse events requiring intensive care unit/critical care unit admission (>1 organ dysfunction)	0 (0.0%)	0 (0.0%)	–
Grade IVb. Adverse events requiring intensive care unit/critical care unit admission (single organ dysfunction)	0 (0.0%)	0 (0.0%)	–
Grade V. Death	0 (0.0%)	2 (20.0%)	–
	*N* = 6	*N* = 10	
Significant adverse events (>grade 2 AGREE)	4 (9.8%)	9 (24.3%)	0.13
Type of adverse events			0.08
Bleeding	3 (42.3%)	2 (15.4%)	–
Perforation/stent dislodgment	1 (14.3%)	3 (23.0%)	–
Stent obstruction	2 (28.6%)	5 (38.5%)	–
Bacteremia	1 (14.3%)	1 (7.7%)	–
Other	0 (0.0%)	2 (15.5%)	–
	*N* = 7	*N* = 13	
Early adverse events (<24 h)			
Early adverse events	4 (9.8%)	5 (13.5%)	0.37
Significant early adverse events (>grade 2 AGREE)	3 (7.3%)	3 (8.1%)	0.99
Type of early adverse event			0.91
Bleeding	2 (50.0%)	1 (10.0%)	–
Perforation/stent dislodgment	1 (25.0%)	2 (40.0%)	–
Acute stent obstruction	0 (0.0%)	0 (0.0%)	–
Bacteremia	1 (25.0%)	1 (20.0%)	–
Other	0 (0.0%)	1 (20.0%)	–
	*N* = 4	*N* = 5	
Severity of early adverse events (AGREE classification)			0.21
Grade I. Adverse events without the need for pharmacologic treatment or endoscopic, radiologic, or surgical interventions	0 (0.0%)	1 (20.0%)	–
Grade II. Adverse events requiring pharmacologic treatment with drugs other than those allowed for grade I adverse events	2 (50.0%)	1 (20.0%)	–
Grade IIIa. Adverse events requiring endoscopic or radiologic intervention	1 (25.0%)	1 (20.0%)	–
Grade IIIb. Adverse events requiring surgical intervention	0 (0.0%)	0 (0.0%)	–
Grade IVa. Adverse events requiring intensive care unit/critical care unit admission (>1 organ dysfunction)	1 (25.0%)	1 (25.0%)	–
Grade IVb. Adverse events requiring intensive care unit/critical care unit admission (single organ dysfunction)	0 (0.0%)	0 (0.0%)	–
Grade V. Death	0 (0%)	1 (20.0%)	–
	*N* = 4	*N* = 5	
Late adverse events (>24 h)			
Late adverse events (>24 h)	3 (7.5%)	8 (21.6%)	0.04
Significant late adverse events (>grade 2 AGREE)	3 (7.5%)	8 (21.6%)	0.04
Type of delayed adverse events			0.40
Bleeding	1 (33.3%)	1 (12.5%)	–
Stent dislodgment	0 (0.0%)	1 (12.5%)	–
Stent obstruction	2 (66.7%)	5 (62.5%)	–
Other	0 (0.0%)	1 (12.5%)	–
	*N* = 3	*N* = 8	–
Severity of delayed adverse events (AGREE classification)			0.13
Grade I. Adverse events with without the need for pharmacologic treatment or endoscopic. radiologic. or surgical interventions	0 (0.0%)	0 (0.0%)	–
Grade II. Adverse events requiring pharmacologic treatment with drugs other than those allowed for grade I adverse events	0 (0.0%)	1 (12.50%)	–
Grade IIIa. Adverse events requiring endoscopic or radiologic intervention	3 (100.0%)	6 (75.0%)	–
Grade IIIb. Adverse events requiring surgical intervention	0 (0.0%)	1 (12.50%)	–
Grade IVa. Adverse events requiring intensive care unit/critical care unit admission (>1 organ dysfunction)	0 (0.0%)	0 (0.0%)	–
Grade IVb. Adverse events requiring intensive care unit/critical care unit admission (single organ dysfunction)	0 (0.0%)	0 (0.0%)	–
Grade V. Death	0 (0.0%)	0 (0.0%)	–
	*N* = 3	*N* = 8	
LAMS dysfunction (immediate and delayed)			
LAMS dysfunction	5 (12.2%)	8 (21.62%)	0.36
Classification of LAMS dysfunctions			
1 Sump syndrome	0 (0.0%)	2 (25.0%)	0.44
2a Sludge or stone impaction	2 (40.0%)	2 (25.0%)	–
2b Food impaction	2 (40.0%)	0 (0.0%)	–
3a LAMS invasion/compression on biliary side	0 (0.0%)	1 (12.5%)	–
3b LAMS invasion/compression on duodenal side	0 (0.0%)	1 (12.5%)	–
4 Migration	1 (20.0%)	2 (25.0%)	–
5 Gastric outlet obstruction	0 (0.0%)	0 (0.0%)	–
	*N* = 5	*N* = 8	
Outcomes after drainage			
Surgery after drainage	4 (9.8%)	4 (11.4%)	0.99
Type of surgery			0.99
Cephalic duodenopancreatectomy	3 (100.0%)	2 (66.7%)	–
Total pancreatectomy	0 (0.0%)	1 (33.3%)	–
	*N* = 3	*N* = 3	
Chemotherapy	18 (47.4%)	21 (58.3%)	0.48
Death	15 (39.5%)	15 (44.1%)	0.87

EUS‐CDS, endoscopic ultrasound‐guided choledochoduodenostomy; EUS‐GBD, endoscopic ultrasound‐guided gallbladder drainage; LAMS, lumen‐apposing metal stent.

#### Technical success

Technical success of the procedures was comparable between both groups: 41/41 (100%) in the EUS‐GBD group vs. 35/37 (94.6%) in the EUS‐CDS group (*P* = 0.132).

#### Adverse events

The occurrence of significant adverse events was observed in 4/41 (9.76%) patients in the EUS‐GBD group and 9/37 (24.32%) patients in the EUS‐CDS group (*P* = 0.128). Notably, bleeding was seen in 3/7 (42.3%) patients in the EUS‐GBD group compared with 2/13 (15.38%) in the EUS‐CDS group. Stent obstruction occurred in 2/7 (28.57%) patients in the EUS‐GBD group and 5/13 (38.46%) in the EUS‐CDS group (*P* = 0.083). Early adverse events occurred in 4/41 (9.8%) patients in the EUS‐GBD group and 5/37 (13.5%) in the EUS‐CDS group (*P* = 0.368). The types of these early adverse events were comparable between both groups (*P* = 0.907).

##### Late adverse events

Late adverse events were more frequent in the EUS‐CDS group, occurring in 8/37 (21.6%) patients, compared with 3/41 (7.3%) in the EUS‐GBD group (*P* = 0.042). For more information on the types and severity of these late adverse events, additional details are provided in Table 3. During follow‐up, LAMS dysfunctions were noted in 5/41 (12.2%) patients in the EUS‐GBD group and 8/37 (21.62%) in the EUS‐CDS group (*P* = 0.364). The specific causes and classifications of these dysfunctions are detailed in Table 3. Postdrainage surgery was required in a similar proportion of patients in both groups: 4/41 (9.8%) in EUS‐GBD and 4/37 (11.4%) in EUS‐CDS (*P* = 0.99). The types of surgeries and other outcomes, including chemotherapy and mortality rates, are elaborated in Table 3.

##### Severe adverse events

In the EUS‐GBD group, one patient had a LAMS dislodgment within an hour postprocedure, leading to biliary peritonitis and necessitating surgical treatment and intensive care unit admission (AGREE grade IVa). In the EUS‐CDS group, two deaths occurred. The first was due to limited treatment options, influenced by the patient's advanced age and the family's refusal for further intervention after LAMS misplacement into the pancreatic duct. This death was attributed to progression of the disease. The second fatality resulted from LAMS entering the peritoneum, which is unmanageable endoscopically. Percutaneous drainage and external drain placement did not ameliorate the ensuing septic condition from biliary peritonitis.

#### Overall survival and time to recurrent biliary obstruction

**Figure 1:**
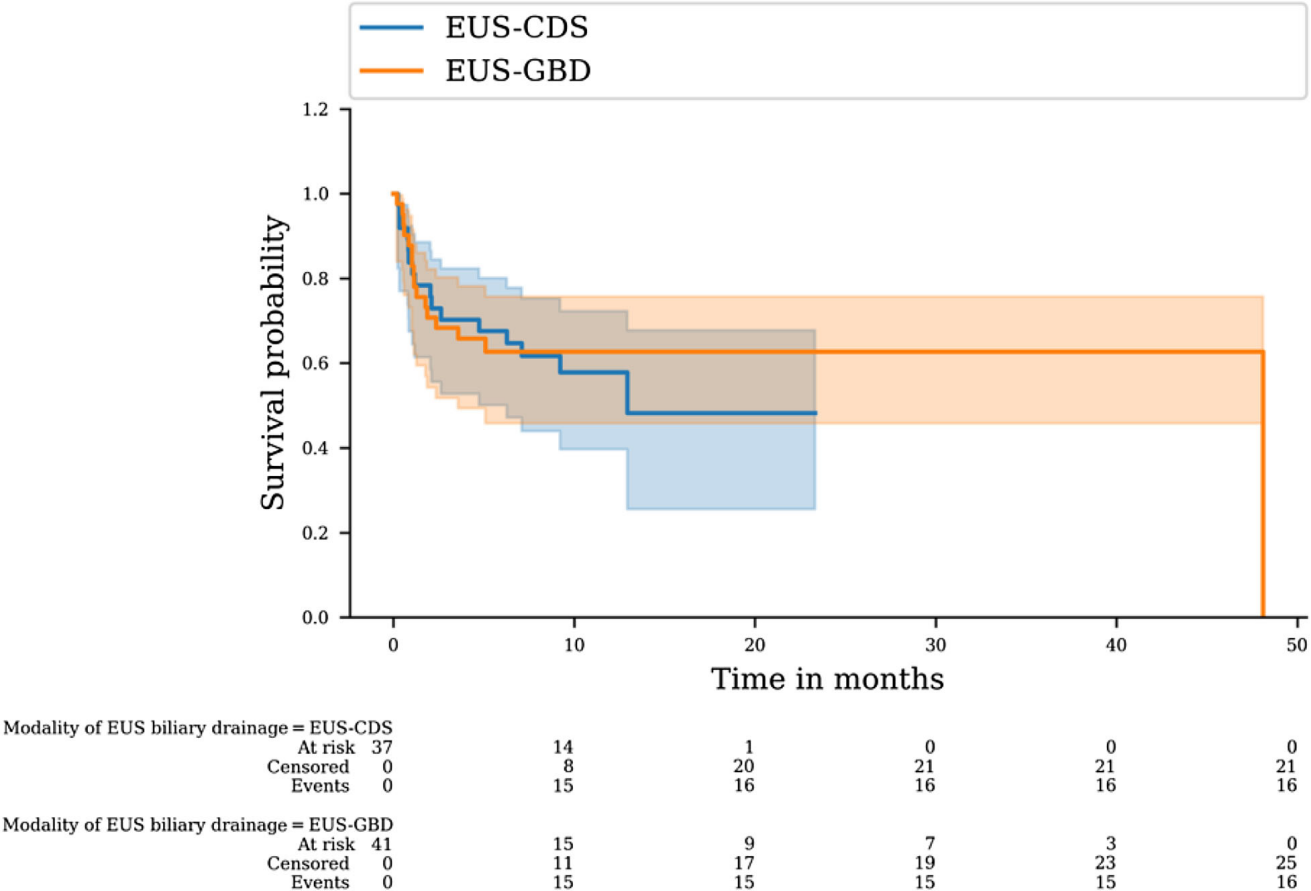
Comparison of overall survival between endoscopic ultrasound‐guided gallbladder drainage (EUS‐GBD) and endoscopic ultrasound‐guided choledochoduodenostomy (EUS‐CDS) groups.

**Figure 2:**
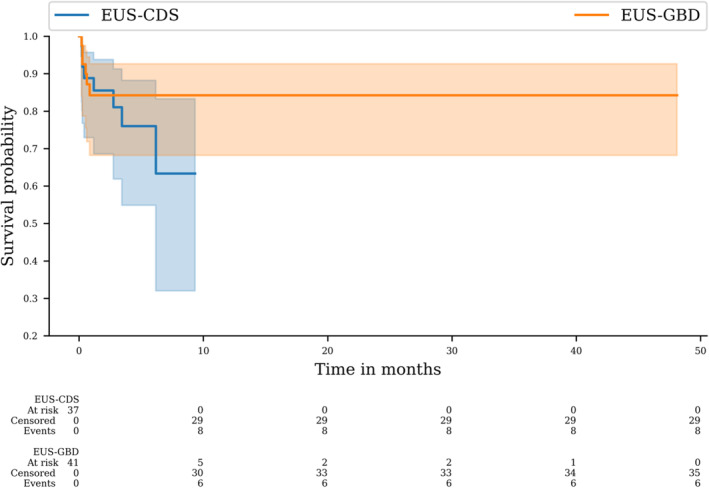
Comparison of biliary patency between endoscopic ultrasound‐guided gallbladder drainage (EUS‐GBD) and endoscopic ultrasound‐guided choledochoduodenostomy (EUS‐CDS) groups.

There was no difference in overall survival distributions for patients in the EUS‐CDS vs. EUS‐GBD groups (log‐rank, *P* = 0.695; Fig. 1). There was also no difference between biliary event‐free survival distributions among patients in the EUS‐CDS vs. EUS‐GBD groups (log‐rank, *P* = 0.541; Fig. 2). No biliary events occurred in the EUS‐GBD group after 26 days. Finally, there was no difference between survival distributions among patients that underwent EUS‐GBD drainage using a 6 × 8, 10 × 10, or 15 × 10 mm LAMS (log‐rank, *P* = 0.709). Among all the deaths, only one was related to the biliary procedure (EUS‐CDS).

#### Subgroup analysis in the population with duodenal stenosis

Both groups demonstrated a high clinical success rate, 16/18 (88.89%) for EUS‐CDS and 15/17 (88.24%) for EUS‐GBD (*P* > 0.999). There were no immediate morbidities observed in either group. However, late adverse events was higher in the EUS‐CDS group 6/18 (33.33%) compared with 1/17 (5.88%) in the EUS‐GBD group, although this difference was not statistically significant (*P* = 0.088). LAMS dysfunction was slightly higher in the EUS‐CDS group at 16.67% vs. 5.88% in the EUS‐GBD group (*P* = 0.603, Table 4).

**Table 4:** Comparison of outcomes between endoscopic ultrasound‐guided gallbladder drainage (EUS‐GBD) and endoscopic ultrasound‐guided choledochoduodenostomy (EUS‐CDS) subgroup analysis in the population with duodenal stenosis

Variable	EUS‐CDS, *N* = 18	EUS‐GBD, *N* = 17	*P*‐value
Clinical success	16 (88.9%)	15 (88.2%)	0.99
Immediate adverse events	0 (0.0%)	0 (0.0%)	0.99
Late adverse events (>24 h)	6 (33.3%)	1 (5.9%)	0.09
LAMS dysfunction	3 (16.7%)	1 (5.9%)	0.60
Severity of delayed adverse events (AGREE classification)			0.13
Grade I. Adverse events with without the need for pharmacologic treatment or endoscopic. radiologic. or surgical interventions	0 (0.0%)	0 (0.0%)	–
Grade II. Adverse events requiring pharmacologic treatment with drugs other than those allowed for grade I adverse events	1 (16.7%)	0 (0%)	–
Grade IIIa. Adverse events requiring endoscopic or radiologic intervention	5 (83.3%)	1 (100.0%)	–
Grade IIIb. Adverse events requiring surgical intervention	1 (16.3%)	0 (0.0%)	–
Grade IVa. Adverse events requiring intensive care unit/critical care unit admission (>1 organ dysfunction)	0 (0.0%)	0 (0.0%)	–
Grade IVb. Adverse events requiring intensive care unit/critical care unit admission (single organ dysfunction)	0 (0.0%)	0 (0.0%)	–
Grade V. Death	1 (0.0%)	0 (0.0%)	–
	*N* = 6	*N* = 1	
Chemotherapy	10 (58.8%)	7 (50.0%)	0.73

LAMS, lumen‐apposing metal stent.

### Follow‐up

The median follow‐up duration was 4.7 (1.21; 48.09) months in the overall population; 5.2 (1.21; 48.09) months in the EUS‐GBD group and 4.0 (2.01; 22.09) months in the EUS‐CDS group.

## DISCUSSION

We observed comparable technical and clinical success between EUS‐GBD and EUS‐CDS in cases of MBDO with failed ERCP, and we found a lower rate of late adverse events in the EUS‐GBD group.

Endoscopic ultrasound‐guided gallbladder drainage has been described as a rescue therapy for MBDO after unsuccessful ERCP or EUS‐CDS in several small, noncomparative, retrospective studies.^17^, ^18^, ^19^ There is only one prospective study on 37 patients with advanced disease that has shown excellent results of EUS‐GBD as a first‐line biliary drainage modality in patients with MBDO in a palliative setting.^20^ This study is the first to compare EUS‐GBD with LAMS to EUS‐CDS with LAMS after failed ERCP in MBDO cases. Additionally, we present the largest cohort of patients who have undergone EUS‐GBD for biliary drainage. Our data appear to indicate that drainage via EUS‐GBD yields comparable rates of clinical success and initiation of chemotherapy; however, the speed of jaundice reduction seems to be slower with this approach.

We found a significantly lower rate of delayed adverse events in the EUS‐GBD group (7.5% vs. 21.6%; *P* = 0.042) and no adverse events occurred after 26 days among patients in the EUS‐GBD group, whereas adverse events occurred progressively over time in the EUS‐CDS group. We noticed two interesting findings in terms of delayed adverse events. First, the type of LAMS dysfunction appeared to be different between the groups. The EUS‐GBD group demonstrated delayed LAMS dysfunction exclusively related to impaction (blood clots or food), whereas the EUS‐CDS group additionally demonstrated dysfunctions related to local invasion at the LAMS side. Second, the time of onset of LAMS dysfunctions was different, with earlier dysfunction in the EUS‐GBD group (<1 month) as opposed to being more spread over time in the EUS‐CDS group. These differences could be explained by several hypotheses. (i) EUS‐GBD is performed far from the tumor site, thus preserving the risk of local invasion or compression at the LAMS side. It is recommended to avoid EUS‐CDS in case of duodenal stenosis for this reason. (ii) The gallbladder space is larger than the CBD after decompression, and this could also preserve this type of drainage from dysfunctions related to LAMS compression on the biliary side by creating a valve effect. Although recommendations advocate for performing EUS‐GBD via duodenal access in cases of cholecystitis,^21^ we chose to perform the procedure via the transgastric route to stay away from the tumoral process and avoid LAMS dysfunction related to duodenal tumor progression. However, it was a deliberate decision on our part to focus solely on the transgastric approach to obtain reliable data on this particular method of conducting the procedure. The transduodenal drainage route is favored in cases of cholecystitis because it is associated with reduced stent migration^22^; however, there are no available data for biliary drainage indications related to tumoral obstructions. Future studies with longer term follow‐up will need to be conducted to determine which approach should be preferred for biliary drainage indications.

The relatively high delayed adverse events rate in our patient series can be explained by two factors. First, we included a high proportion of patients with duodenal stenosis (45% of the patients), which is indeed an independent risk factor for biliary adverse events during follow‐up.^5^, ^23^ Second, the general condition of our patients included was relatively poor because more than 60% of them had an Eastern Cooperative Oncology Group/World Health Organization performance status score ≥2. Finally, EUS‐GBD biliary drainage did not preclude secondary pancreatic surgery. Indeed, three patients underwent surgery following the procedure without any major adverse events.

The diameter of the CBD of patients in the EUS‐GBD group was significantly smaller than patients in the EUS‐CDS group, with 40% of patients with a CBD diameter <12 mm because it is recommended to avoid EUS‐CDS in cases of insufficiently dilated bile ducts.^24^ When EUS‐CDS is risky, EUS‐GBD provides biliary decompression during the same time without needing secondary percutaneous drainage, which has lower success and higher morbidity after failed ERCP compared with EUS drainage.^3^, ^25^, ^26^ However, to ensure that the cystic duct is not tumor‐invaded, EUS‐GBD requires preliminary assessment of cystic duct implantation because 15% of the general population have an intrapancreatic cystic duct insertion.^27^ This can be assessed preoperatively by cross‐sectional imaging, but in our opinion, it should always be confirmed per procedure by EUS. Similarly, our retrospective data collection was unable to capture the presence of gallstones or ultrasonographic signs of cholecystitis during the EUS‐GBD procedure. This could have an impact on biliary patency, although limited data exist in the literature to confirm this.

Technically, our small patient series does not allow us to recommend a LAMS size for EUS‐GBD in MDBO. A 15 × 15 mm LAMS allows easy reintervention but can lead to large food debris deposition, as seen in our two cases. Also, our study only reports on the transgastric route without concluding which route is preferable.

The results of our study should be interpreted with caution because of several limitations inherent to its retrospective design and multicenter nature involving different teams from different tertiary centers. This may have led to loss of data during patient follow‐up, and different centers may have adopted different strategies for managing various situations. To limit the risk of data loss, when possible, we contacted the patients' referring physicians or general practitioners if patients were lost to follow‐up or if patients went on to be managed at different centers. Second, despite the clinical success of EUS‐GBD and EUS‐CDS groups being broadly comparable, the choice of the procedure was a parameter that we could not control. Indeed, this choice was according to the operators' discretion and depended either on unfavorable CBD measurements, presence of duodenal stenosis, or personal technical convenience. Indeed, this is particularly evident in our results because patients in the EUS‐GBD group exhibited a smaller CBD diameter compared with the EUS‐CDS group (13.5 mm vs. 18.2 mm, *P* < 0.001). This finding clearly illustrates how the size of the biliary tract could have influenced the operators' choice of procedure, making the execution of EUS‐CDS potentially more challenging when the CBD is minimally dilated. Although this did not seem to affect the comparability of the groups in our study (same disease severity, duodenal stenosis, ascites, Eastern Cooperative Oncology Group score), future prospective studies with control of indications for EUS‐GBD need to be carried out to define in which contexts this procedure must be preferred and to define which LAMS size to use in which approach (transgastric vs. transduodenal).

Finally, our study has a particular focus on biliary drainage using LAMS; however, in cases of difficult access to the duodenal papilla or duodenal stenosis, drainage via hepaticogastrostomy has been shown to be a viable option and is likely preferable under these conditions.^23^ Nevertheless, this mode of biliary drainage is technically more challenging and has a steeper learning curve, especially when the bile ducts are not dilated. In such instances, EUS‐GBD may be a convenient and safe means for the patient to achieve resolution of the biliary obstruction.

In conclusion, in cases of failed ERCP in patients with MDBO and without previous cholecystectomy, EUS‐GBD should be considered for biliary decompression for cases with either an insufficiently dilated CBD or a >10 mm distance between the duodenal wall and the CBD. The EUS‐GBD procedure overcomes the limitations of the EUS‐CDS procedure and allows for an effective salvage biliary drainage with robust clinical results and high tolerance.

## CONFLICT OF INTEREST

Authors declare no conflict of interest for this article.

## FUNDING INFORMATION

None.
